# The Asymmetric Total Synthesis of the Female-Produced Sex Pheromone of the Tea Tussock Moth

**DOI:** 10.3390/molecules29163866

**Published:** 2024-08-15

**Authors:** Zhi-Feng Sun, Hao Liu, Yi-Fan Li, Yan-Ping Duan, Ling-Xia Jin, Xiao-Hui Ji, Hui-Ping Dai, Jiu-Fu Lu

**Affiliations:** Shaanxi Key Laboratory for Catalysis, College of Chemical and Environment Science, Shaanxi University of Technology, Hanzhong 723001, China; liuh@snut.edu.cn (H.L.); liyf@snut.edu.cn (Y.-F.L.); duanyp@snut.edu.cn (Y.-P.D.); jinlx@snut.edu.cn (L.-X.J.); jixh@snut.edu.cn (X.-H.J.); daihp@snut.edu.cn (H.-P.D.)

**Keywords:** sex pheromone, tea tussock moth, asymmetric synthesis, Julia–Kocienski olefination

## Abstract

The tea tussock moth is a pest that damages tea leaves, affecting the quality and yield of tea and causing huge economic losses. The efficient asymmetric total synthesis of the sex pheromone of the tea tussock moth was achieved using commercially available starting materials with a 25% overall yield in 11 steps. Moreover, the chiral moiety was introduced by Evans’ template and the key C-C bond construction was accomplished through Julia–Kocienski olefination coupling. The synthetic sex pheromone of the tea tussock moth will facilitate the subsequent assessment and implementation of pheromones as environmentally friendly tools for pest management.

## 1. Introduction

**Figure 1:**
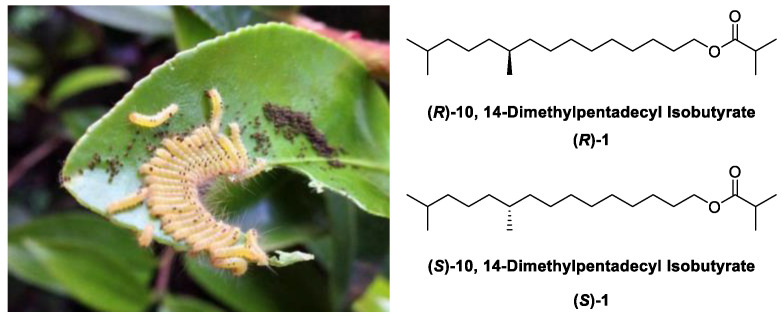
Tea tussock moth and chemical structure of its sex pheromone.

Tea plays a significant role in the agricultural economy of China [1]. However, the infestation of pests on tea plants poses a more severe threat; in particular, the tea tussock moth, *Euproctis pseudoconspersa* (Strand) (Lepidoptera: Lymantridae), is a major pest of tea planation in China. Its larvae consume tea leaves, leading to complete defoliation in severe cases, significantly compromising the quality and yield of tea. Additionally, the venomous hairs of larvae also pose a risk of injury to farmers working in the tea plantations [2]. Currently, the primary means of pest control relies heavily on chemical pesticides, resulting in challenges such as exceeding pesticide residue standards, which has become a hindrance to the development of China’s tea industry. In response to the growing health consciousness among individuals, it is crucial to investigate pollution-free preventive and control measures in tea gardens. Insect pheromones offer numerous advantages, such as their non-toxic nature, sparing of natural predators, and environmental friendliness [3]. Consequently, the utilization of insect pheromones for pest control has attracted significant interest among scientists. The chemical structure of the sex pheromone emitted by the tea tussock moth was identified as 10, 14-dimethyl-l-pentadecyl isobutyrate **1** (Figure 1) [4,5]. The naturally occurring pheromone of the tea tussock moth was preliminarily assigned to the (*R*)-configuration at the C-10 methyl group [6]. However, field trials revealed that both the (*R*)-enantiomer and (*S*)-enantiomer exhibit attractive properties [7,8].

**Scheme 1:**
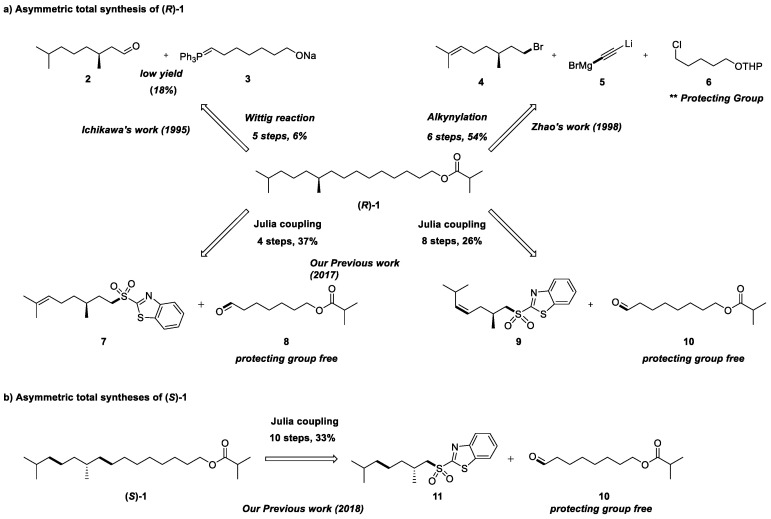
Structure and asymmetric total syntheses of (*R*)- and (*S*)-**1** [6,8,11,12].

Insect pheromones are commonly classified as either chiral or achiral compounds, characterized by their low molecular weight and simple functional groups, typically esters [9]. The synthesis of insect pheromones heavily relies on the construction of carbon chains, for which various synthetic strategies exist based on different raw materials [10]. The synthesis of the sex pheromone of the tea tussock moth in the early literature often relied primarily on alkylation methods [5]. There are two main problems including the construction of the C-C skeleton and the utilization of expensive chiral starting materials. For instance, Ichikawa et al. reported the first asymmetric total synthesis of the sex pheromone of the tea tussock moth by employing a Wittig reaction strategy using commercially available (*R*)- and (*S*)-citronellol (Scheme 1) [6]. Zhao and co-workers reported the second total synthesis employing an alkylation strategy by using commercially available (*R*)*-* and (*S*)-citronellyl bromide (Scheme 1a) [8]. Our group have reported the total synthesis of the sex pheromone of (*R*)-**1** and (*S*)-**1** through a Julia–Kocienski olefination coupling strategy (Scheme 1) [11,12].

As for the Julia–Kocienski reaction [13], sulfone as a key compound can be more readily synthesized through the Mitsunobu reaction [14] and oxidation reactions from easily available simple alcohols. Therefore, the Julia–Kocienski reaction seems more convenient and environmentally friendly. Given this, as a continuation of our interest in organic synthesis, we still hope to develop another new route using Julia–Kocienski olefination to achieve the construction of the C-C skeleton using diverse starting materials. We herein report an asymmetric synthetic approach for the total synthesis of (*R*)-**1**.

## 2. Results and Discussion

**Scheme 2:**
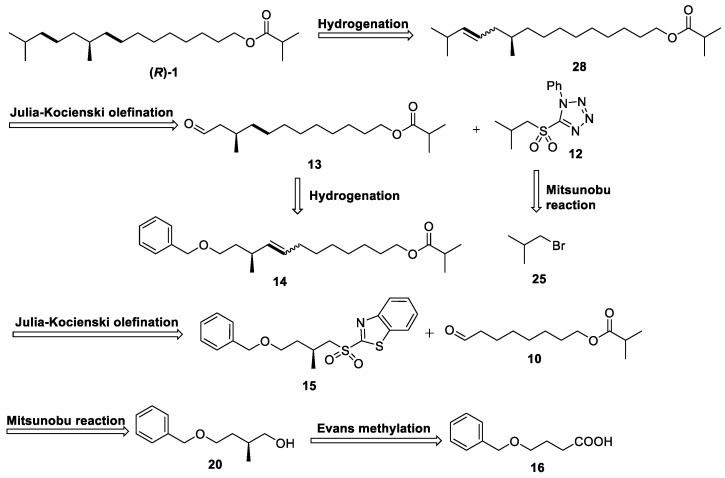
Retrosynthesis of sex pheromone of (*R*)-**1**.

The retrosynthesis of (*R*)-**1** is shown in Scheme 2. The target molecule (*R*)-**1** can be readily obtained from alkene **28** through catalytic hydrogenation reduction. The synthesis of alkene **28** can be achieved through Julia–Kocienski olefination between PT-sulfone **12** and chiral aldehyde **13**. The synthesis of PT-sulfone **12** can be accomplished by isobutyl bromide **25** in a two-step process involving the Mitsunobu reaction [14] and subsequent oxidation. Chiral aldehyde **13** can be synthesized from olefin **14** via both catalytic hydrogenation and oxidation processes. Olefin **14** was synthesized via Julia–Kocienski olefination using BT-sulfone **15** and aldehyde **10** as starting materials. BT-sulfone **15** can still be synthesized from chiral alcohol **20** through a two-step process involving the Mitsunobu reaction and oxidation. The synthesis of chiral alcohol **20** can be accomplished via a four-step process starting from 4-benzyloxybutyric acid **16** [15], with the pivotal step being the asymmetric chiral induction using Evans’ template [16].

**Scheme 3:**
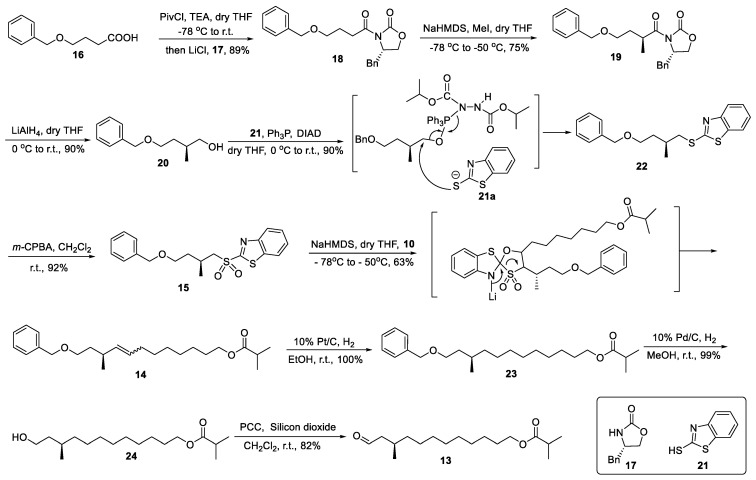
Synthesis of key intermediates of **13**.

The synthesis of the key intermediates of **13** is shown in Scheme 3. The compound **18** was synthesized by reacting Evans’ template **17** with acid **16** in the presence of piavloyl chloride [17], triethylamine, and LiCl [18], resulting in a yield of 89%. This suggests that LiCl exhibits commendable catalytic activity in the acylation reaction, and the role of LiCl is to form intermediates with a complex structure with the components participating in the reaction. The desired compound **19** was obtained in 75% yield through deprotonation of **18** with NaHMDS, followed by highly diastereoselective alkylation of the resulting chiral imide enolate with MeI at a low temperature. Due to the highly efficient alkali catalytic properties of NaHMDS, its reaction activity and deprotonation ability are enhanced at elevated temperatures. However, conducting the reaction at a low temperature is advantageous for achieving a higher yield of the target product **19** while minimizing the formation of undesired byproducts [19]. The enantiomeric excess (ee) of the product **19** was determined to be 99% using high-performance liquid chromatography (HPLC) with a chiral AD-H column. Subsequently, compound **19** was treated with LiAlH_4_ in dry THF to afford the desired primary alcohol **20**, which possessed a chiral center, in a yield of 90%. Next, the chiral alcohol **20** was effectively converted into thioether **22** in the presence of PPh_3_ and DIAD via the Mitsunobu reaction with MBT **21** in a 90% yield [14]. The mechanism of the Mitsunobu reaction is illustrated in Scheme 3. Initially, triphenylphosphine (PPh_3_) undergoes a reaction with diisopropyl azodicarboxylate (DIAD), leading to the formation of an active intermediate. This intermediate subsequently abstracts a proton from mercaptan **21**, while alcohol **20** attacks the phosphine cationic center to generate phosphine oxide, and simultaneously, DIAD dissociates as a byproduct of hydrazine. Subsequently, the anions of the nucleophilic mercaptan **21** initiate a reverse attack on the alkylphosphine oxide intermediate, resulting in the formation of the product **22** and the concomitant generation of triphenylphosphine oxide as a byproduct. The enantioselectivity of Evans methylation was determined to be 91:9 *er* for the derivative **20** through high-performance liquid chromatography (HPLC) analysis on a chiral OD-H column.

Thioether **22** was further oxidized with *m*-CPBA to afford sulfone **15** in a high yield of 92% [20]. Meanwhile, the aldehyde **10** was synthesized following the established protocol in [11] using 1, 8-octanediol through a two-step procedure. Subsequently, Julia–Kocienski olefination of aldehyde **10** with sulfone **15** deprotonated by NaHMDS resulted in the formation of alkene **14** as a mixture of *Z*/*E* (3:1, determined from ^1^H NMR) isomers, with a yield of 63%. Julia–Kocienski olefination [13] proceeds through a five-membered ring transition state, as depicted in Scheme 3. Then, alkene **14** was followed by hydrogenation to give compound **23** in a 100% yield using a Pt/C catalyst to avoid racemization of the allylic methyl group [21]. The compound **23** was subjected to Pd/C catalytic hydrogenation removal of the benzyl protecting group, giving **24** in a 99% yield. The compound **24** was oxidized by PCC dispersed with silicon dioxide to obtain the key intermediate aldehyde **13** with a yield of 82%. Because the addition of silicon dioxide can enhance the adsorption of the byproduct resulting from PCC, the oxidation of compound **24** was better promoted by PCC. This strategy not only enhances reaction efficiency but also mitigates environmental pollution through byproduct adsorption [22].

**Scheme 4:**
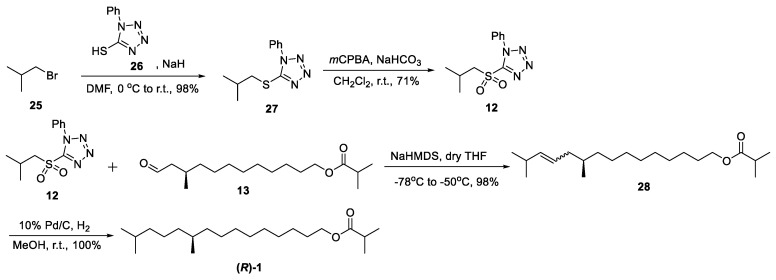
Synthesis of sex pheromone of (*R*)-**1**.

As outlined in Scheme 4, isobutyl bromide **25** was converted into sulfone **12** via *S*-alkylation and *m*-CPBA/NaHCO_3_ oxidation in dry CH_2_Cl_2_ in a 70% yield over two steps. For the total synthesis of (*R*)-**1**, compound **28** was first completed using sulfone **12** and chiral aldehyde **13** via Julia–Kocienski olefination in 98% yields, and then further subjected to Pd/C catalytic hydrogenation, giving the target molecule (*R*)-**1** in a100% yield. All of the data are consistent with the literature values [4,5].

## 3. Experimental Section

### 3.1. General Method

All commercially available reagents were used without further purification. THF and diethyl ether were distilled from sodium under argon. Dichloromethane was distilled from calcium hydride under argon. Column chromatography was performed on silica gel (200–400 mesh). The optical rotations were measured using a polarimeter equipped with a sodium lamp. ^1^H NMR (500 MHz, TMS at δ 0.00 ppm or CDCl_3_ at δ 7.26 ppm) and ^13^C NMR (125 MHz, CDCl_3_ at δ 77.00 ppm as internal standard) spectra were recorded on a Bruker 500 MHz NMR spectrometer. HRMS data were recorded on Thermo Scientific LTQ Orbitrap XL (Waltham, MA, USA).

### 3.2. (S)-4-Benzyl-3-(4-(benzyloxy)butanoyl)oxazolidin-2-one *(**18**)*

A stirred solution of **16** (1.5 g, 7.7 mmol) in anhydrous THF (38 mL) under argon was cooled to −78 °C for 15 min, and then added with Et_3_N (2.1 mL, 15.4 mmol) followed by PivCl (1.1 mL, 9.3 mmol). After being stirred for 20 min at −78 °C, the mixture was warmed to room temperature and stirred for 1 h. Solid LiCl (0.66 g, 15.62 mmol) and (*S*)-4-benzyloxazolidin-2-one (**17**) (1.4 g, 7.7 mmol) were added at −78 °C. The reaction mixture was stirred for 1 h at −78 °C, slowly warmed to room temperature, and stirred overnight. The reaction was quenched with H_2_O (10 mL), and the mixture was extracted with ethyl acetate (2 × 10 mL). The combined organic layers were washed with brine (30 mL), dried over anhydrous Na_2_SO_4_, and concentrated under reduced pressure. The crude product was purified using silica gel (EtOAc/hexane 1:5) to give compound **18** as a colorless oil (2.4 g, 89%). ^1^H NMR (500 MHz, CDCl_3_) δ 7.34–7.31 (m, 6H), 7.28–7.25 (m, 2H), 7.18 (d, *J* = 7.1 Hz, 2H), 4.63–4.58 (m, 1H), 4.50 (s, 2H), 4.13–4.08 (m, 2H), 3.58 (t, *J* = 6.2 Hz, 2H), 3.26 (dd, *J* = 3.3, 13.4 Hz, 1H), 3.08–3.05 (m, 2H), 2.69 (dd, *J* = 9.7, 13.4 Hz, 1H), 2.06–2.01 (m, 2H); ^13^C NMR (125 MHz, CDCl_3_) δ 173.04, 153.45, 138.44, 135.35, 129.37, 128.91, 128.33, 127.67, 127.53, 127.28, 72.88, 69.23, 66.12, 55.14, 37.87, 32.46, 24.48.

### 3.3. (S)-4-Benzyl-3-((S)-4-(benzyloxy)-2-methylbutanoyl)oxazolidin-2-one *(**19**)*

A stirred solution of **18** (2.4 g, 6.9 mmol) in anhydrous THF (30 mL) at −78 °C under argon was added to a solution of NaHMDS (2.0 M in THF, 6.9 mL, 13.7 mmol). After being stirred at −78 °C for 30 min, MeI (0.83 mL, 13.42 mmol) was added dropwise and then stirring was continued for another 2 h at −78 °C. Then, the reaction was allowed to warm to −50 °C and stirred overnight. The reaction mixture was quenched with saturated NH_4_Cl solution (10 mL), and the mixture was warmed to room temperature and then extracted with ethyl acetate (2 × 30 mL). The combined organic layers were washed with brine (30 mL), dried over anhydrous Na_2_SO_4_, and concentrated under reduced pressure. The crude product was purified using silica gel (EtOAc/hexane 1:5) to give compound **19** as a colorless oil (1.9 g, 75%). [α]^23^_D_ = +45.531 (*c* = 2.6, CHCl_3_). ^1^H NMR (500 MHz, CDCl_3_) δ 7.32–7.20 (m, 8H), 7.14 (d, *J* = 7.0 Hz, 2H), 4.42 (d, *J* = 2.7 Hz, 2H), 4.0–3.92 (m, 2H), 3.73 (t, *J* = 8.6 Hz, 1H), 3.59–3.51 (m, 2H), 3.18 (dd, *J* = 3.3, 13.4 Hz, 1H), 2.7 (dd, *J* = 9.5, 13.4 Hz, 1H), 2.21–2.14 (m, 1H), 1.77–1.71 (m, 1H), 1.26–1.2 (d, *J* = 6.9 Hz, 3H); ^13^C NMR (125 MHz, CDCl_3_) δ 177.03, 153.20, 138.50, 135.40, 129.35, 128.78, 128.21, 127.58, 127.46, 127.17, 72.78, 68.43, 65.79, 55.15, 37.95, 35.11, 33.61, 18.01.

### 3.4. (S)-4-(Benzyloxy)-2-methylbutan-1-ol *(**20**)*

A stirred solution of LiAlH_4_ (2.7 g, 71.4 mmol) in anhydrous THF (55 mL) at 0 °C under argon was added dropwise to a mixture of **19** (5.2 g, 14.3 mmol) and anhydrous THF (40 mL). The reaction mixture was allowed to warm to room temperature, stirred overnight, and quenched with H_2_O, 10% NaOH solution, andH_2_O (1:2:3) at 0 °C. The mixture was filtered under reduced pressure and the resulting residue was washed with ethyl acetate. The aqueous layer was extracted with ethyl acetate (2 × 20 mL). The combined organic layers were dried with Na_2_SO_4_ and concentrated under reduced pressure. The crude product was purified using silica gel (EtOAc/hexane 1:10) to afford compound **20** as a colorless oil (2.5 g, 90%). [α]^23^_D_ = +4.113 (*c* = 2.3, CHCl_3_). ^1^H NMR (500 MHz, CDCl_3_) δ 7.36–7.27 (m, 5H), 4.52 (s, 2H), 3.61–3.57 (m, 1H), 3.55–3.48 (m, 2H), 3.43 (dd, *J* = 6.6, 10.9 Hz, 1H), 2.62 (s, 1H), 1.85–1.78 (m, 1H), 1.74–1.67 (m, 1H), 1.60–1.54 (m, 1H), 0.92 (d, *J* = 6.9 Hz, 3H); ^13^C NMR (125 MHz, CDCl_3_) δ 137.99, 128.40, 127.71, 127.67, 73.10, 68.64, 68.03, 34.06, 33.97, 29.65, 17.13.

### 3.5. (S)-2-(4-(Benzyloxy)-2-methylbutylthi-o) benzo[d]thiazole *(**22**)*

A solution of **20** (1.9 g, 10.0 mmol), PPh_3_ (3.1 g, 12.0 mmol), and **21** in THF (50 mL) was added to DIAD (2.4 mL, 12.0 mmol) at 0 °C. The reaction mixture was allowed to warm to room temperature, stirred for 4 h, and then concentrated under reduced pressure. The crude product was purified using silica gel (EtOAc/hexane 1:30) to afford compound **22** as a yellow oil (3.1 g, 90%). [α]^23^_D_ = +7.305 (*c* = 3.0, CHCl_3_). ^1^H NMR (500 MHz, CDCl_3_) δ 7.84 (d, *J* = 8.1 Hz, 1H), 7.74 (d, *J* = 8.0 Hz, 1H), 7.41–7.83 (m, 1H), 7.32–7.27 (m, 6H), 4.51 (d, *J* = 3.1 Hz, 2H), 3.62–3.54 (m, 2H), 3.46 (dd, *J* = 5.6, 12.8 Hz, 1H), 3.25 (dd, *J* = 7.4, 12.9 Hz, 1H), 2.2–2.12 (m, 1H), 1.92–1.85 (m, 1H), 1.65–1.61 (m, 1H), 1.09 (d, *J* = 6.8 Hz, 3H); ^13^C NMR (125 MHz, CDCl_3_) δ 167.60, 153.32, 138.45, 135.20, 128.41, 127.68, 127.59, 126.02, 124.13, 121.46, 120.94,73.01, 68.13, 40.58, 35.74, 30.70, 19.39.

### 3.6. (S)-2-(4-(Benzyloxy)-2-methylbutylsulfonyl)benzo[d]thiazole *(**15**)*

A solution of **22** (2.7 g, 8.0 mmol) in CH_2_Cl_2_ (80 mL) at room temperature was added to *m*-CPBA (70% purity, 6.9 g, 40.0 mmol). The reaction mixture was stirred at room temperature overnight, quenched with the saturated Na_2_S_2_O_3_ aqueous solution (10 mL), stirred for 10 min, neutralized with the saturated NaHCO_3_ solution (10 mL), extracted with CH_2_Cl_2_ (3 × 10 mL), washed with brine (20 mL), dried over anhydrous Na_2_SO_4,_ and concentrated under reduced pressure. The crude product was purified using silica gel (EtOAc/hexane 1:10) to give compound **15** as a yellowish oil (1.9 g, 92%). ^1^H NMR (500 MHz, CDCl_3_) δ 8.2 (d, *J* = 8.3 Hz, 1H), 8.0 (d, *J* = 7.9 Hz, 1H), 7.65–7.57 (m, 2H), 7.43–7.23 (m, 5H), 4.41 (s, 2H), 3.72 (dd, *J* = 4.5, 14.4 Hz, 2H), 3.55–3.48 (m, 2H), 3.39 (dd, *J* = 8.2, 14.4 Hz, 1H), 2.53–2.47 (m, 1H), 1.87–1.8 (m, 1H), 1.69–1.62 (m, 1H), 1.18 (d, *J* = 6.8 Hz, 3H); ^13^C NMR (125 MHz, CDCl_3_) δ 166.72, 152.74, 138.18, 136.80, 128.40, 128.0, 127.66, 127.62, 125.46, 122.40, 73.0, 67.53, 60.67, 36.21, 26.53, 20.02. HRMS (ESI): *m*/*z* calculated for C_16_H_24_NO_2_S_2_^+^ (M + H)^+^: 326.12430; found: 326.12448.

### 3.7. (S, E)-12-(Benzyloxy)-10-methyldodec-8-enyl isobutyrate *(**14**)*

A solution of **15** (0.8 g, 2.1 mmol) in anhydrous THF (25 mL) under argon was added to NaHMDS (2.0 M in THF, 1.6 mL, 3.2 mmol), the reaction mixture was stirred at −78 °C for 30 min, and a solution of 8-oxooctyl isobutyrate **10** (0.7 g, 3.2 mmol) in anhydrous THF (25 mL) was added dropwise. The reaction mixture was stirred at −78 °C for 3 h, slowly warmed to −50 °C, and stirred overnight. The reaction mixture was quenched with a saturated NH_4_Cl aqueous solution at −50 °C, the mixture was warmed to room temperature, and the aqueous layer was extracted with ethyl acetate (2 × 5 mL), washed with brine (20 mL), dried over anhydrous Na_2_SO_4_, and concentrated under reduced pressure. The crude product was purified using silica gel (EtOAc/hexane 1:200) to give compound **14** as a colorless oil (0.5 g, 63%). (*Z*/*E* 3:1 mixture) [α]^23^_D_ = +3.056 (*c* = 2.5, CHCl_3_). ^1^H NMR (500 MHz, CDCl_3_) δ 7.33–7.25 (m, 5H), 5.36–5.08 (m, 2H), 4.49–4.44 (m, 2H), 4.06–4.03 (m, 2H), 3.49–3.39 (m, 2H), 2.65–2.5 (m, 2H), 2.07–1.93 (m, 2H), 1.7–1.43 (m, 4H), 1.3 (s, 8H), 1.16 (d, *J* = 7 Hz, 6H), 0.98–0.95 (m, 3H); ^13^C NMR (125 MHz, CDCl_3_) δ 177.25, 138.76, 138.73, 135.53, 129.05, 128.34, 127.66, 127.61, 127.47, 72.99, 68.82, 64.4, 37.28, 34.08, 29.85, 29.25, 29.19, 28.68, 28.61, 27.43, 25.93, 25.9, 21.51, 19.05.

### 3.8. (R)-12-(Benzyloxy)-10-methyldodecyl isobutyrate *(**23**)*

A suspension of 10% Pt on carbon (104 mg, 0.5 mmol) and **14** (503 mg, 1.3 mmol) in ethanol (45 mL) were pretreated with hydrogen by five vacuum–hydrogen cycles. The mixture was stirred at room temperature under a hydrogen balloon for 12 h, the catalyst was filtered off, and the solvent was removed under reduced pressure. The crude product was purified using silica gel (EtOAc/hexane 1:50) to give compound **23** as a colorless oil (495 mg, 100%). [α]^23^_D_ = +1.761 (*c* = 2.6, CHCl_3_). ^1^H NMR (500 MHz, CDCl_3_) δ 7.34–7.26 (m, 5H), 4.5 (s, 2H), 4.05 (t, *J* = 6.7 Hz, 2H), 3.52–3.47 (m, 2H), 2.57–2.51 (m, 1H), 1.69–1.56 (m, 4H), 1.45–1.38 (m, 1H), 1.33–1.27 (m, 14H), 0.86 (d, *J* = 6.6 Hz, 3H); ^13^C NMR (125 MHz, CDCl_3_) δ 177.29, 138.76, 128.36, 127.63, 127.48, 72.92, 68.81, 64.43, 37.15, 36.82, 34.09, 29.94, 29.9, 29.61, 29.55, 29.28, 28.69, 26.97, 25.94, 21.51, 19.71, 19.06. HRMS (ESI): *m*/*z* calculated for C_24_H_41_O_3_^+^ (M + H)^+^: 377.30502; found: 377.30499.

### 3.9. (R)-12-Hydroxy-10-methyldodecyl isobutyrate *(**24**)*

A suspension of 10% Pd on carbon (104 mg, 0.5 mmol) and (*R*)-12-(benzyloxy)-10-methyldodecyl isobutyrate (**23**) (503 mg, 1.3 mmol) in ethanol (45 mL) were pretreated with hydrogen by five vacuum–hydrogen cycles. The mixture was stirred at room temperature under a hydrogen balloon for 12 h, the catalyst was filtered off, and the solvent was removed under reduced pressure. The crude product was purified using silica gel (EtOAc/hexane 1:10) to give compound **24** as a colorless oil (495 mg, 99%). ^1^H NMR (500 MHz, CDCl_3_) δ 4.06 (t, *J* = 6.7 Hz, 2H), 3.7–3.65 (m, 2H), 2.53 (td, *J* = 5, 10 Hz, 1H), 1.63–1.6 (m, 4H), 1.39–1.27 (m, 15H), 1.16 (d, *J* = 5.0 Hz, 6H), 0.89 (d, *J* = 5.0 Hz, 3H); ^13^C NMR (125 MHz, CDCl_3_) δ 177.31, 64.42, 61.26, 40.0, 37.14, 34.08, 29.88, 29.56, 29.51, 29.5, 29.24, 28.66, 26.94, 25.9, 19.66, 19.03. HRMS (ESI): *m*/*z* calculated for C_17_H_35_O_3_^+^ (M + H)^+^: 287.25807; found: 287.25818.

### 3.10. 5-(Isobutylthio)-1-phenyl-1H-tetrazole *(**27**)*

A solution of NaH (2.7 g, 83.3 mmol) in DMF (70 mL) at 0 °C was added to a solution of **25** (8.9 g, 83.3mmol) in DMF (30 mL). The reaction mixture was stirred at 0 °C for 10 min, and **26** (5.4 mL, 83.3 mmol) was added and stirred for 12 h. The reaction mixture was quenched with water, extracted with ethyl acetate, washed with brine, dried over anhydrous Na_2_SO_4,_ and concentrated under reduced pressure. The crude product was purified using silica gel (EtOAc/hexane 1:5) to give compound **27** as a colorless oil (13.0 g, 98%). ^1^H NMR (500 MHz, CDCl_3_) δ 7.64–7.58 (m, 5H), 3.35 (d, *J* = 6.9 Hz, 2H), 2.18–2.1 (m, 1H), 1.1 (d, *J* = 6.7 Hz, 6H); ^13^C NMR (125 MHz, CDCl_3_) δ 154.75, 133.8, 130.1, 129.8, 123.93, 41.81, 28.35, 21.74.

### 3.11. 5-(Isobutylsulfonyl)-1-phenyl-1H-tetrazole *(**12**)*

A solution of **27** (12.8 g, 54.2 mmol) in CH_2_Cl_2_ (424 mL) was added to NaHCO_3_ (22.8 g, 270.9 mmol) at room temperature. The reaction mixture was stirred at room temperature for 5 min, and *m*-CPBA (22.3 g, 90.4 mmol) was added and stirred overnight. The reaction mixture was added to a Na_2_S_2_O_3_ solution (10 mL) and a saturated NaHCO_3_ aqueous solution (15 mL), stirred for 30 min, extracted with CH_2_Cl_2_, washed with brine, dried over anhydrous Na_2_SO_4,_ and concentrated under reduced pressure. The crude product was purified using silica gel (EtOAc/hexane 1:5) to give compound **12** as a yellowish oil (10.3 g, 71%). ^1^H NMR (500 MHz, CDCl_3_) δ 7.68–7.6 (m, 5H), 3.68 (d, *J* = 6.6 Hz, 2H), 2.53–2.45 (m, 1H), 1.16 (d, *J* = 6.8 Hz, 6H); ^13^C NMR (125 MHz, CDCl_3_) δ 133.13, 131.47, 129.71, 125.18, 63.11, 23.97, 22.55.

### 3.12. (R, E)-10, 14-Dimethylpentadec-12-enyl isobutyrate *(**28**)*

A solution of **12** (269 mg, 1.0 mmol) in anhydrous THF (8 mL) under argon at −78 °C was slowly added to a solution of NaHMDS (2.0 M in THF, 0.5 mL, 1.0 mmol) dropwise. After stirring at −78 °C for 30 min, a solution of **13** (190 mg, 0.7 mmol) in anhydrous THF (8 mL) was added dropwise and stirred for another 2 h at −78 °C. The reaction mixture was allowed to warm to −50 °C and stirred overnight. The reaction mixture was quenched with a saturated NH_4_Cl solution (5 mL) at −50 °C, and the mixture was warmed to room temperature, extracted with ethyl acetate (2 × 10 mL), washed with brine (10 mL), dried over anhydrous Na_2_SO_4,_ and concentrated under reduced pressure. The crude product was purified using silica gel (EtOAc/hexane 1:5) to give compound **28** as a colorless oil (213 mg, 98%). [α]^23^_D_ = +1.997 (*c* = 2.3, CHCl_3_). ^1^H NMR (500 MHz, CDCl_3_) δ 5.35–5.21 (m, 2H), 4.05 (d, *J* = 6.8 Hz, 2H), 2.59–2.5 (m, 1H), 2.26–2.22 (m, 1H), 2.04–1.76 (m, 2H), 1.63–1.59 (m, 2H), 1.31–1.25 (m, 15H), 1.16 (d, *J* = 7 Hz, 6H), 0.95 (dd, *J* = 6.8, 15 Hz, 6H), 0.84 (dd, *J* = 6.7, 12.6 Hz, 3H); ^13^C NMR (125 MHz, CDCl_3_) δ 177.3, 138.86, 138.17, 126.04, 125.69, 64.44, 40.04, 36.73, 34.64, 34.09, 33.22, 29.93, 29.61, 29.54, 29.27, 28.68, 27.18, 27.07, 26.48, 25,93, 23.18, 23.14, 22.78, 19.05.

### 3.13. (R)-10, 14-Dimethylpentadecyl isobutyrate ((R)-***1***)

A suspension of 10% Pd on carbon (13 mg, 0.04 mmol) and **28** (6.5 mg, 0.05 mmol) in ethanol (10 mL) were pretreated with hydrogen using five vacuum–hydrogen cycles. The mixture was stirred under a hydrogen balloon at room temperature for 12 h, the catalyst was filtered off, and the solvent was removed under reduced pressure. The crude product was purified using silica gel (EtOAc/hexane 1:5) to give compound (*R*)-**1** as a colorless oil (13 mg, 100%). [α]^23^_D_ = +0.581 (c = 2.3, CHCl_3_). ^1^H NMR (500 MHz, CDCl_3_) δ 4.05 (t, *J* = 6.8 Hz, 2H), 2.56–2.5 (m, 1H), 1.65–1.48 (m, 4H), 1.4–1.19 (m, 17H), 1.16 (d, *J* = 7 Hz, 6H), 1.15–1.04 (m, 3H), 0.85 (dd, *J* = 6.6, 13.2 Hz, 9H); ^13^C NMR δ (125 MHz, CDCl_3_) 176.08, 63.16, 38.37, 36.32, 36.09, 33.05, 31.76, 28.97, 28.59, 28.51, 28.24, 27.66, 26.97, 26.06, 24.9, 23.79, 21.7, 21.61, 18.7, 18.0. HRMS (ESI): *m*/*z* calculated for C_21_H_43_O_2_^+^ (M + H)^+^: 327.32576; found: 327.32599.

## 4. Conclusions

In summary, we have successfully achieved the asymmetric total synthesis of (*R*)-**1** with a 25% overall yield in 11 steps, a major component of the sex pheromone of the tea tussock moth. The C-C skeletons were constructed by employing Julia–Kocienski coupling as the key step, while Evans’ template was utilized for asymmetric-induced methylation. The concise, efficient synthetic route to (*R*)-**1** will facilitate the subsequent evaluation and application of pheromones as environmental benign tools for pest management.

## Data Availability

The data presented in this article are available in the text.

## References

[1] Li Z.-Q., Yuan T.-T., Cui S.-W., Zhao Y.-J., Shao Y.-H., Shang J.-N., Luo Z.-X., Cai X.-M., Bian L., Chen Z.-M. (2023). Development of a high-efficiency sex pheromone formula to control. J. Integr. Agric..

[2] Wang X.-Q., Gu Q.-Y., Zhang W., Jiang H.-Y., Chen S.-C., Smagghe G., Niu J.-Z., Wang J.-J. (2021). Prevalence of a novel bunyavirus in tea tussock moth *Euproctis pseudoconspersa* (Lepidoptera: Lymantriidae). J. Insect Sci..

[3] Wang T.-K., Liu X.-F., Luo Z.-X., Cai X.-M., Li Z.-Q., Bian L., Xiu C.-L., Chen Z.-M., Li Q.-R., Fu N.-X. (2024). Transcriptome-wide identification of cytochrome P450s in tea black tussock moth (*Dasychira baibarana*) and candidate genes involved in Type-II sex pheromone biosynthesis. Insects.

[4] Wakamura S., Yasuda T., Ichikawa A., Fukumoto T., Mochizuki F. (1994). Sex attractant pheromone of the tea tussock moth, *Euproctis pseudoconspersa* (Strand) (Lepidoptera: Lymantriidae): Identification and field attraction. Appl. Entomol. Zool..

[5] Zhao C.-H., Millar J.G., Wen Z., Wang S., Wang X., Zhu Y. (1996). Isolation, identification, and synthesis of the female sex pheromone of the tea tussock moth, *Euproctis pseudoconspersa* (Lepidoptera: Lymantridae). Entomol. Sin..

[6] Ichikawa A., Yasuda T., Wakamura S. (1995). Absolute configuration of sex pheromone for tea tussock moth, *Euproctis pseudoconspersa* (Strand) via synthesis of (*R*)- and (*S*)-10, 14- dimethyl-1-pentadecyl isobutyrates. J. Chem. Ecol..

[7] Wakamura S., Ichikawa A., Yasuda T., Arakaki N., Fukumoto T. (1996). EAG and field responses of the male tea tussock moth, *Euproctis pseudoconspersa* (Strand) (Lepidoptera: Lymantriidae) to (*R*)- and (*S*)-enantiomers and racemic mixture of 10, 14-dimethylpentadecyl isobutyrate. Appl. Entomol. Zool..

[8] Zhao C.-H., Millar J.G., Pan K.-H., Xu C.-S. (1998). Responses of tea tussock moth, *Euproctis pseudoconspersa*, to its pheromone, (*R*)-10, 14-dimethylpentadecyl isobutyrates, and to the *S*-enantiomer of its pheromone. J. Chem. Ecol..

[9] Müller M., Buchbauer G. (2011). Essential oil components as pheromones. A review. Flavour Fragr. J..

[10] Zarbin P.H.G., Villar J.A.F.P., Corrêa A.G. (2007). Insect Pheromone Synthesis in Brazil: An Overview. J. Braz. Chem. Soc..

[11] Sun Z.-F., Zhou L.-N., Meng Y., Zhang T., Du Z.-T., Zheng H. (2017). Concise asymmetric synthesis of the sex pheromone of the tea tussock moth. Tetrahedron Asymmetry.

[12] Zhang H.-L., Sun Z.-F., Zhou L.-N., Liu L., Zhang T., Du Z.-T. (2018). Synthesis of the sex pheromone of the tea tussock moth based on a resource chemistry strategy. Molecules.

[13] Blakemore P.R. (2002). The modified Julia olefination: Alkene synthesis via the condensation of metallated heteroarylalkylsulfones with carbonyl compounds. J. Chem. Soc. Perkin Trans..

[14] Mitsunobu O. (1981). The use of diethyl azodicarboxylate and triphenylphosphine in synthesis and transformation of natural products. Synthesis.

[15] Braun M., Atalick S., Guldi D.M., Lanig H., Brettreich M., Burghardt S., Hatzimarinaki M., Ravanelli E., Prato M., Eldik R.V. (2003). Electrostatic complexation and photo induced electron transfer between Zn-Cytochrome *c* and polyanionic fullerene dendrimers. Chem. Eur. J..

[16] Evans D.A., Ennis M.D., Mathre D.J. (1982). Asymmetric alkylation reactions of chiral imide enolates. A practical approach to the enantioselective synthesis of α -substituted carboxylic acid derivatives. J. Am. Chem. Soc..

[17] Yadav J.S., Thirupathaiah B., Ghamdi A.A.K.A. (2012). Protecting group free formal total synthesis of the antitubercular agent erogorgiaene. Eur. J. Org. Chem..

[18] Liu D.B., Qiu Z.M., Liu Z.W. (2008). Research progress on catalysis of Lithium chloride. Inorg. Chem. Ind..

[19] Yang Z.C., Jin X.M., Guaciaro M., Molino B.F. (2012). Asymmetric Synthesis and Absolute Configuration of Streptophenazine G. J. Org. Chem..

[20] Summeren R.P.V., Moody D.B., Feringa B.L., Minnaard A.J. (2006). Total synthesis of enantiopure *β*-D-mannosyl phosphomycoketides from *Mycobacterium tuberculosis*. J. Am. Chem. Soc..

[21] Li N.-S., Scharf L., Adams E.J., Piccirilli J.A. (2013). Highly stereocontrolled total synthesis of *β*-D-mannosyl phosphomycoketide: A natural product from *Mycobacterium tuberculosis*. J. Org. Chem..

[22] Corey E.J., Suggs J.W. (1975). Pyridinium chlorochromate. Efficient reagent for oxidation of primary and secondary alcohols to carbonyl compounds. Tetrahedron Lett..

